# Association of left atrial pressure with late gadolinium enhancement extent in patient who underwent catheter ablation for atrial fibrillation

**DOI:** 10.1038/s41598-020-72929-0

**Published:** 2020-10-05

**Authors:** Seung-Young Roh, Dae In Lee, Sung Ho Hwang, Kwang-No Lee, Yong-soo Baek, Mohammad Iqbal, Dong-Hyeok Kim, Jinhee Ahn, Jaemin Shim, Jong-Il Choi, Young-Hoon Kim

**Affiliations:** 1 Division of Cardiology, Department of Internal Medicine, Korea University College of Medicine and Korea University Guro Hospital, 148, Gurodong-ro, Guro-gu, Seoul, 08308 Republic of Korea; 2 Division of Cardiology, Department of Internal Medicine, Chungbuk National University Hospital, 776, 1 Sunhwan-ro, Seowon-gu, Cheongju-si, Chungcheongbuk-do 28644 Republic of Korea; 3 Department of Radiology, Korea University College of Medicine and Korea University Anam Hospital, 73 Inchon-ro, Seongbuk-gu, Seoul, 02841 Republic of Korea; 4 Division of Cardiology, Department of Internal Medicine, Ajou University Hospital, 164 World Cup-ro, Yeongtong-gu, Suwon, 16499 Republic of Korea; 5 Division of Cardiology, Department of Internal Medicine, Inha University Hospital, 27, Inhang-ro, Jung-gu, Incheon, 22332 Republic of Korea; 6 Division of Cardiology, Department of Internal Medicine, Dr. Hasan Sadikin Central General Hospital, University Padjadjaran, Bandung, West Java 40161 Indonesia; 7 Division of Cardiology, Department of Internal Medicine, Ewha Womans University Seoul Hospital, 260, Gonghang-daero, Gangseo-gu, Seoul, 07804 Republic of Korea; 8 Division of Cardiology, Department of Internal Medicine, Pusan National University Hospital, 179 Gudeok-ro, Amidong 1-ga, Seo-gu, Busan, 49241 Republic of Korea; 9 Division of Cardiology, Department of Internal Medicine, Korea University College of Medicine and Korea University Anam Hospital, 73 Inchon-ro, Seongbuk-gu, Seoul, 02841 Republic of Korea

**Keywords:** Magnetic resonance imaging, Cardiology, Interventional cardiology, Arrhythmias

## Abstract

Atrial remodeling with fibrosis has been well-described in patients with atrial fibrillation (AF). We hypothesized that the left atrial (LA)-late gadolinium enhancement (LGE) extent on cardiac magnetic resonance (CMR) imaging is associated with LA pressure and can be a marker for suitable candidates for non-paroxysmal AF ablation. A total of 173 AF patients with an LA-LGE area on CMR imaging were enrolled. The clinical parameters, including invasively measured LA pressure, were compared between the patients with extensive LA-LGE (E-LGE, LGE extent ≥ 20%, n = 78) and those with small LA-LGE (S-LGE, LGE extent < 20%, n = 95). The E-LGE group had higher peak LA pressures than the S-LGE group (23 versus 19 mmHg, p < 0.001). The E-LGE group had more patients with non-paroxysmal AF (non-PAF) (51% vs. 34%), heart failure (9% vs. 0%), and higher NT pro-B-type natriuretic peptide (472 vs. 265 pg/ml) (all p < 0.05). LA pressure ≥ 21 mmHg was an independent predictor of E-LGE (OR = 2.218; p = 0.019). In the paroxysmal AF (PAF) subgroup, freedom from atrial arrhythmia after catheter ablation was not different (81% vs 86%, log-rank p = 0.529). However, in the non-PAF subgroup, it was significantly higher in the S-LGE group than in the E-LGE group (81% vs 55%, log-rank p = 0.014). Increased LA pressure was related to the LA-LGE extent. LA-LGE was a good predictor of outcome after catheter ablation, but only in patients with non-PAF.

## Introduction

Atrial fibrillation (AF) is the most common sustained cardiac arrhythmia, with prevalence increasing along with the age of the population^1^. The clinical consequences of AF are increased risks of cardiac morbidity and mortality driven by stroke and congestive heart failure (HF), and it is associated with decreased quality of life^2,3^. The catheter ablation of AF is an important treatment modality for patients with drug-refractory AF. However, the reported success rates of AF ablation vary widely, ranging from 40 to 80%^4^. Despite recent developments in mapping and ablation tools, ablation fails to maintain long-term, sinus rhythm in a substantial number of patients. The guidelines for AF treatment remain controversial, and better patient selection criteria for ablation are needed. Atrial fibrosis is a determinant in the success of rhythm control strategies, including catheter ablation^5,6^, and is a marker of atrial remodeling and considered a cause of AF perpetuation. Identification of left atrial (LA) fibrosis is crucial to deciding on the optimal treatment strategy for AF. The role of cardiac magnetic resonance (CMR) has received attention for selecting the best candidates for ablation^7^.

Late gadolinium enhancement (LGE) CMR is a non-invasive imaging technique that assesses the structure and tissue composition of the heart. However, the clinical role of CMR imaging in patients with AF has not yet been fully established. The atrial wall myocardium is thin, and irregular ventricular rhythms complicate functional analysis during AF. Furthermore, the implications and formative processes of LA-LGE remain to be elucidated.

We sought to evaluate the association between LA pressure and the LA-LGE extent in CMR images in AF ablation candidates. The clinical value of LA-LGE on CMR imaging as a predictor of atrial arrhythmia recurrence was investigated in patients who underwent catheter ablation.

## Methods

### Study population

We prospectively enrolled consecutive patients referred for catheter ablation for drug-refractory AF. Only patients without a previous ablation for AF and without contraindications for CMR were included. LGE-CMR, transthoracic echocardiography (TTE), and transesophageal echocardiography (TEE) were performed within 24 h prior to catheter ablation. All participants provided written informed consent. The clinical type of AF was categorized as paroxysmal AF (PAF, AF that self-terminated within 7 days) or non-PAF (AF lasting more than 7 days or needing cardioversion for termination).

**Figure 1:**
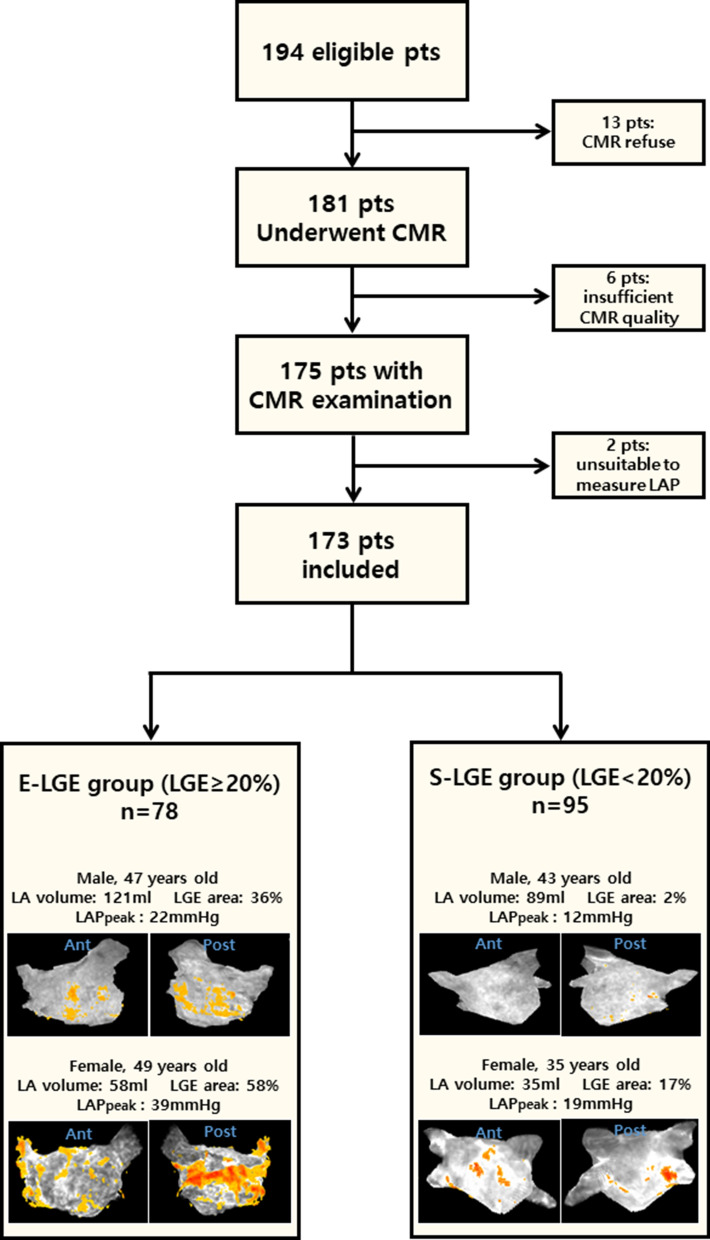
Flow chart showing the study design and representative LGE-CMR images of the E-LGE and S-LGE groups. *Pts* patients, *CMR* cardiac magnetic resonance, *LAP* left atrial pressure, *LGE* late gadolinium enhancement, *LA* left atrial, *LAP* left atrial pressure, *Ant* anterior, *Post* posterior.

The study population was divided into two groups based on the percentage of LA-LGE. The LGE area was calculated as described below. The extensive LGE (E-LGE) group included patients with an LGE extent ≥ 20%, of the mean LA-LGE value of all patients and the small LGE (S-LGE) group included those with LGE extents < 20%. We compared the clinical and functional parameters between the two groups to investigate the pathogenesis of LA-LGE. The study design flow chart and a representative case from both groups are shown in Fig. 1. In this study, six patients were excluded because of unacceptable quality of CMR images among the patients underwent CMR. During the LGE image acquisition, the mean heart rate of all of the patients was maintained below 100 beats per minute.

This study was performed in accordance with the ethical standards of the 1964 Declaration of Helsinki. The study protocol was approved by the Institutional Review Board of Korea University Medical Center.

### LGE CMR protocol

The LA-LGE CMR protocol and the LGE quantification method were described in our previously reported study^8,9^. CMR was performed using a 3-T MR system (ACHIEVA; Philips Medical Systems, Best, The Netherlands) with a 32-element phased-array cardiac coil. MR angiography of the pulmonary veins (PVs) was obtained using a contrast-enhanced timing robust angiography sequence after an injection of 0.2 mmol/kg gadolinium-based contrast (DOTAREM; Guerbet, S.A., Villepinte, France). High spatial resolution LGE-CMR images were acquired approximately 20 min after the injection of the contrast agent using a 3-dimensional (3D) inversion recovery-prepared, respiration-navigated, electrocardiography (ECG)-gated, gradient-echo pulse sequence after the Look-Locker sequence to identify the optimal nulling time for the normal left ventricular myocardium. The typical acquisition parameters of the LGE-CMR images were as follows: voxel size, 1.5 mm × 1.5 mm × 1.5 mm; repetition time/echo time, 4.7/1.4 ms; inversion time, 230–270 ms; flip angle, 25°; bandwidth, 127 Hz/pixel; one RR interval between the inversion pulses; phase encoding direction in the right-left; and a parallel imaging technique using sensitivity encoding with R = 2. All LGE-CMR images were transferred into the workstation for quantitative analysis.

### Analysis of LGE-CMR images

The LGE-CMR images were analyzed using Terarecon iNtuition software (TeraRecon, Foster City, CA, USA) by a radiologist (SHH) who was blinded to the LA pressure and other clinical information. On each coronal or transverse LGE-CMR image, the endocardial and epicardial borders of the LA wall were semi-automatically contoured to select the LA wall. A 3D LA model was reconstructed by combining the entire LA wall voxels. In the LA wall, the voxels of enhancement were identified using the full width at half-maximum (FWHM) technique with a manual delineation of the regions of interest (ROI) focusing on the mitral valve leaflet. The ROI was drawn around the mitral valve leaflet with high signal intensity to define the maximum signal for the FWHM technique, which provided the signal threshold as one-half of the maximum signal from the mitral valve leaflet. The LA wall pixels with signal intensities greater than the signal threshold of LGE were automatically selected, and defined as the LA-LGE pixels. After the identification of the LA-LGE pixels over the entire slices, a color look-up table mask was applied yellow to the LA-LGE pixels and the white to other pixels on the 3D LA model for better delineation of the LA wall composition. To assist in the process, the initial visualization used a volume-rendering tool in CORVIEW (Marrek Inc) that allowed the distribution of enhancement in 3D. Then, the LA wall voxels of enhancement were defined as LA-LGE. The sum of the LA-LGE over all of the LGE-CMR images was considered the LA-LGE volume (in ml). Furthermore, the extent of LA-LGE was calculated as the percentage of the LA-LGE volume in the total LA wall volume (Fig. S1).

### Functional analysis

ECG-gated cine CMR was performed to evaluate the LA function. The cine CMR images were analyzed according to a previously published method^8,9^. Briefly, a radiologist assessed the LA areas on the perpendicular planes of the 2- and 4-chamber CMR images. The LA axis length from the mitral annulus level to the posterior wall of the LA was evaluated in the 2-chamber cine CMR images. The biplane area-length technique for the calculation of LA volume was based on this formula: LA volume (ml) = (0.85 × LA area on the 4-chamber cine CMR image × LA area on the 2-chamber CMR image)/LA axis length in the 2-chamber cine CMR image. We calculated the LA volumes based on the LA areas and LA axis length was determined from the cine CMR images. The LA volume measurements were indexed to the body surface area. The left ventricular (LV) ejection fraction (EF), E/Em ratio, and right ventricular systolic pressure were measured by TTE. The presence of spontaneous echo contrast and thrombus was evaluated by TEE.

### Biomarkers

Blood samples were collected from the peripheral vein of each patient within 24 h prior to the LGE-CMR. NT pro-B-type natriuretic peptide (BNP), von Willebrand factor (vWF), D-dimer, and high-sensitivity C-reactive protein (hs-CRP) were measured to evaluate their relationships with the LA-LGE extent. We compared the biomarkers between the E-LGE and S-LGE groups.

### Measurements of LA pressure

All patients fasted 8 h prior to catheter ablation. Saline fluid (0.9%) was injected during that time at a rate of 40 ml/h. Sedation was used during the procedure and pressure measurements. The LA was assessed via transseptal puncture using a long sheath (SCHWARTZ LEFT 1, St. Jude Medical, Minnetonka, MN, USA) (Fig. S2A). The LA pressure was measured during the sinus rhythm (SR) using a 6-Fr pigtail catheter (Cordis Corp, Warren, NJ, USA) immediately after a transseptal puncture. If the baseline rhythm was AF, the measurement was repeated 5 min later after restoring SR by internal (2–10 J) or external (100–200 J) cardioversion. If the patient’s breathing was unstable, the pressure measurement was based on the inspiration state. The LA pressure was measured when the sinus rate was between 60 and 100 bpm. When the LA pressure varied more than 5 mmHg during respiration, we used the median value of the LA pressure between inspiration and expiration. LA pressure was defined as the height of the v wave during SR (Fig. S2B). The investigators who measured the LA pressure were blinded to the quantification of the LGE in the CMR images.

### Catheter ablation

The circumferential antral ablation of 4-PVs was performed guided by a NavX 3-dimensional mapping system (St. Jude Medical, USA). The endpoint of PV ablation was eliminating all PV potentials. The radiofrequency energy was delivered with a power of 30 W and a target temperature of 48 ℃ (Irvine Biomedical, Inc, CA, USA) using a 4-mm open irrigated-tip catheter (COOLFLEX; St. Jude Medical). The subsequent strategy was different according to clinical type of AF. The procedural endpoint was eliminating all triggers, in addition to PV isolation (PVI) in patients with PAF. That endpoint in patients with non-PAF was termination and no further induction of sustained atrial arrhythmia. If AF was not terminated after PVI, LA complex fractionated atrial electrograms (CFAEs) were mapped. If AF was organized to AT during PVI or CFAE ablation, activation mapping-guided ablation was performed. If SR was not restored after the ablation of the aforementioned lesions, direct-current electrical cardioversion was used.

### Follow-up

Follow-up rhythm examinations and 12-lead ECGs were performed at 1, 3, 6, 9, and 12 months after the procedure. Holter or 30-day event recorder monitoring was performed at 3, 6, and 12 months. Recurrence as a clinical outcome was defined as any episode of AF/AT lasting more than 30 s after a blanking period of 3 months. Antiarrhythmic drugs (AADs) were continued for 1–3 months after the procedure and discontinued at the end of the blanking period.

### Statistical analysis

Continuous variables are expressed as the mean plus or minus the standard deviation. Categorical variables are reported as counts with percentages. An independent Student’s *t* test was used to compare the continuous variables between the E-LGE and S-LGE groups, and the categorical variables were compared by Chi-squared test or Fisher’s exact test. Multivariate analysis was conducted with a logistic regression model reporting odds ratios (OR). The predictor variables included age, female, AF type, diagnosis to ablation time, body mass index, CHA_2_DS_2_ VASc ≥ 2, LA pressure ≥ 21 mmHg, the LA volume index, LV EF, NT pro-BNP, and E/Em ratio. Multiple regression analysis was performed using the criterion of p < 0.10 in univariate analysis for the inclusion of a variable in the model. The time to AF/AT recurrence was measured using Kaplan–Meier analysis with comparisons by log-rank statistics. A p-value of less than 0.05 was considered significant statistically. SPSS 20.0 software (SPSS Inc., Chicago, IL, USA) was used for all statistical analyses.

## Results

### Baseline characteristics

Between December 2014 and September 2015, 194 consecutive patients with drug-refractory AF were referred for catheter ablation of AF. None had a history of AF procedures or thoracic surgery, and none had significant mitral valve disease. After excluding 13 patients who declined consent or could not undergo CMR, 181 patients underwent TTE, TEE, and LGE-CMR within 24 h prior to ablation. In this study, six patients were excluded because of unacceptable quality of CMR images among the patients underwent CMR. During the LGE image acquisition, the mean heart rate of all of the patients was maintained below 100 beats per minute. One patient with cardiac tamponade before measurement of the LA pressure and another patient who failed AF cardioversion were excluded. Ultimately, 173 patients (mean age 57 ± 10, males 81%, non-PAF 42%) were included in the analysis. The LA-LGE area measured by CMR imaging showed a maximum of 58% and a mean of 19%. The E-LGE group contained 78 patients (45%), and the S-LGE group contained 95 patients (55%). A comparison of the baseline characteristics between the groups is provided in Table 1. Age, gender, body mass index, and the time from diagnosis to ablation were not different between the groups. More patients in the E-LGE group had non-PAF compared to patients in the S-LGE group (51% vs. 34%, p = 0.021). The clinical type of AF did not correlate well with the LGE extent in each group. Twenty-three (33%) patients with non-PAF were included in the S-LGE group, whereas 23 (42%) patients with PAF had E-LGE. The patients with non-PAF (n = 72) in the total population did not have significantly higher LA-LGE compared to the patients with PAF (n = 101) (21 ± 11% vs. 18 ± 11%, p = 0.108). The E-LGE group had significantly more frequent history of HF (9% vs. 0%, p = 0.003).

**Table 1:** Baseline characteristics of study population.

	E-LGE group (n = 78)	S-LGE group (n = 95)	*p* value
Age (years)	57 ± 10	56 ± 11	0.540
Male gender	62 (80)	78 (82)	0.403
Body mass index (kg/m^2^)	26 ± 5	25 ± 3	0.099
Non-paroxysmal AF	40 (51)	32 (33)	0.021
Diagnosis to ablation time (month)	43 ± 34	46 ± 46	0.655
**Comorbidity**			
Hypertension	27 (35)	38 (40)	0.285
Diabetes mellitus	5 (6)	9 (10)	0.328
Thromboembolism	6 (8)	8 (8)	0.545
History of heart failure	7 (9.0)	0	0.003
Coronary artery disease	4 (5)	5 (5.3)	0.622
Chronic kidney disease	3 (4)	3 (3.2)	0.562
Thyroid disease	5 (6)	7 (7)	0.525
CHADS2 VASc ≥ 2	29 (37)	32(34)	0.374
**CMR data**			
LGE area (%)	29 ± 8	11 ± 5	< 0.001
LA volume (ml)	96 ± 34	87 ± 29	0.091
LA volume index (ml/m^2^)	54 ± 20	49 ± 17	0.085
**Echocardiographic data**			
LV mass index (g/m^2^)	87 ± 16	91 ± 16	0.113
LV ejection fraction (%)	57 ± 6	58 ± 4	0.465
E/Em ratio	8.5 ± 2.5	9.2 ± 4.0	0.192
Estimated PASP (mmHg)	28 ± 7	26 ± 8	0.265
Spontaneous echo contrast	20 (25.6)	16 (16.8)	0.109
**Measured LA pressure**			
LAP_peak_ (mmHg)	23 ± 6	19 ± 5	< 0.001
LAP_nadir_ (mmHg)	9 ± 4	8 ± 4	0.006
LAP_mean_ (mmHg)	14 ± 4	12 ± 4	< 0.001

Data are presented as n (%) or mean ± standard deviation and analyzed using χ^2^ test and unpaired two-tailed t-test or Fisher’s exact test.

*AF* atrial fibrillation, *CMR* cardiac magnetic resonance, *LGE* late gadolinium enhancement, *LA* left atrial, *LV* left ventricular, *PASP* pulmonary artery systolic pressure, *LAP* left atrial pressure.

### LA structure and function

The LA anatomical data and function were analyzed by CMR imaging and echocardiography (Table 1). The mean LA-LGE was significantly greater in the E-LGE group than in the S-LGE group (29 ± 8% vs. 11 ± 5%, p < 0.001). The LA volume (LAV, 96 ± 34 ml vs. 87 ± 29 ml, p = 0.091) and LAV index (LAVI, 54 ± 20 vs. 49 ± 17 ml/m^2^, p = 0.085) tended to be higher in the E-LGE group than in the S-LGE group. The LV EF (57 ± 6 vs. 58 ± 4%, p = 0.465) and E/Em ratio (9 ± 3 vs. 9 ± 4, p = 0.192) measured by TTE were not different between the two groups. The existence of spontaneous echo contrast was not different between the groups.

### LA pressure and extent of LA-LGE

**Figure 2:**
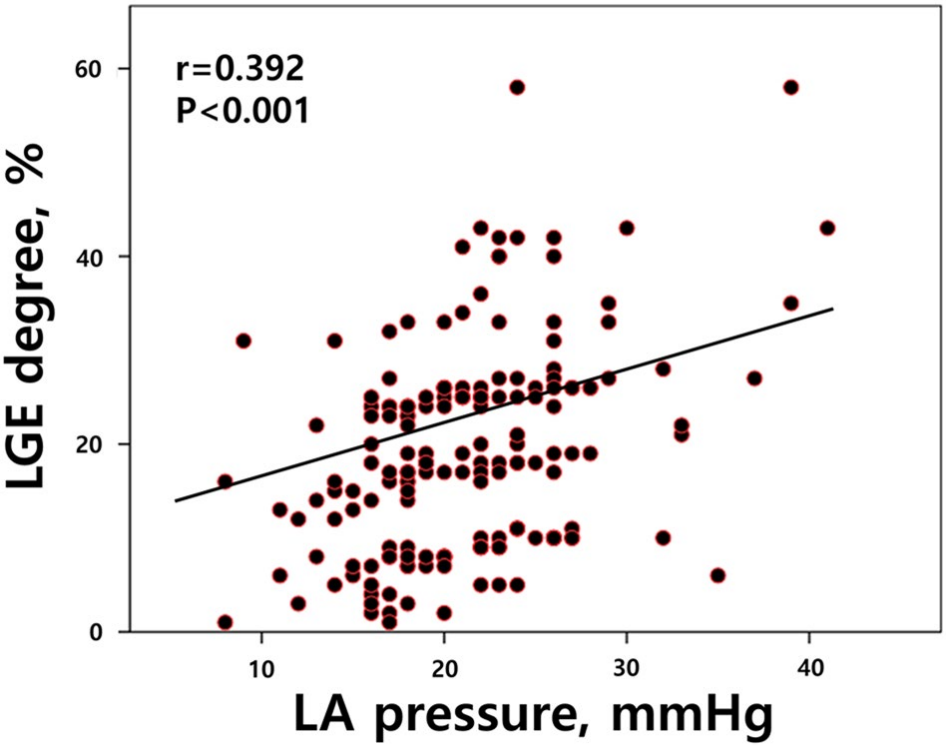
The relationship between measured left atrial pressure and late gadolinium enhancement (LGE) extent in cardiac magnetic resonance. Each point on the plots represents a value of the LGE extent in one patient. R and P represent Pearson's correlation coefficients and the corresponding P-values. *LA* left atrial, *LGE* late gadolinium enhancement, *LA pressure* left atrial pressure.

The LA pressure measurements were compared between the groups. It was significantly higher in the E-LGE group compared to the S-LGE group (23 ± 6 vs. 19 ± 5 mmHg, p < 0.001) (Table 1). Increased LA pressure correlated with higher LGE (correlation analysis, R = 0.392, p < 0.001). (Fig. 2). Univariate and multivariate logistic regression analyses were performed to determine the predictors of E-LGE (Table 2). LA pressure ≥ 21 (OR = 2.218; 95% CI 1.138–4.324; p = 0.019) was the only independent predictor of E-LGE.

**Table 2:** Univariate and multivariate analysis (Logistic regression) showing odds ratio to predict E-LGE (LGE ≥ 20%).

Variables	Univariate odds ratio (95% CI)	*p* value	Multivariate odds ratio (95% CI)	*p* value
Age, 1 year	1.009 (0.980–1.039)	0.538		
Female sex	1.184 (0.554–2.531)	0.663		
Non-paroxysmal AF	2.072 (1.120–3.833)	0.020	1.835 (0.899–3.747)	0.096
Diagnosis to ablation time (month)	0.998 (0.991–1.006)	0.998		
Body mass index (kg/m^2^)	1.077 (0.986–1.177)	0.100	1.063 (0.964–1.171)	0.222
CHADS2 VASc score ≥ 2	1.165 (0.623–2.179)	0.632		
LV ejection fraction (%)	0.978 (0.922–1.038)	0.978		
E/Em ratio	0.937 (0.843–1.041)	0.223		
LA volume index (ml/m^2^)	1.015 (0.998–1.032)	0.082	1.009 (0.989–1.029)	0.399
NT Pro BNP (pg/ml)	1.001 (1.000–1.002)	0.025	1.001 (1.000–1.002)	0.157
LA pressure ≥ 21 mmHg	2.235 (1.213–4.118)	0.010	2.218 (1.138–4.324)	0.019

*AF* atrial fibrillation, *LV* left ventricular, *LA* left atrial, *NT Pro BNP* N terminal brain natriuretic peptides.

### Biomarkers

Four biomarkers were measured in all enrolled patients. NT Pro B-type natriuretic peptide (NT pro-BNP) was significantly greater in the E-LGE group (472 ± 729 pg/ml vs. 264 ± 303 pg/ml, p = 0.021) (Table 1 and Fig. S3). Von Willebrand factor (vWF) (125 ± 49% vs. 126 ± 52%, p = 0.916), d-dimer (0.37 ± 0.50 μg/ml vs. 0.29 ± 0.14 μg/ml, p = 0.132), and C-reactive protein (CRP) (12 ± 91 vs. 2 ± 7, p = 0.356) were not different between the groups.

### Clinical outcomes

**Figure 3:**
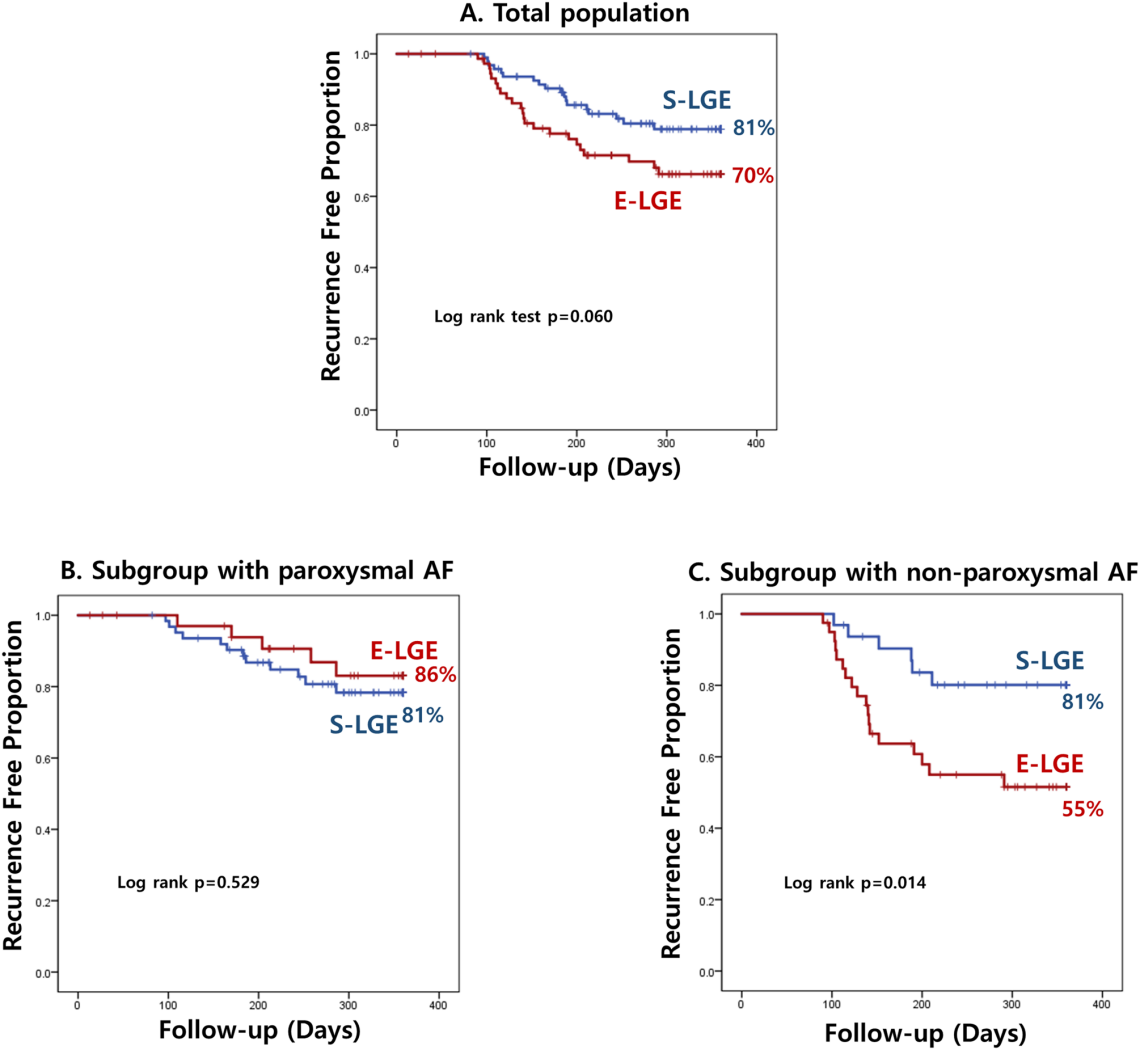
Kaplan–Meier survival curve showing atrial fibrillation (AF)/atrial tachycardia (AT)-free survival with the proportion of patients in sinus rhythm against the follow-up time in days. (**A**) AF/AT-free survival in the total population, (**B**) AF/AT-free survival in the subgroup with paroxysmal AF, (**C**) AF/AT-free survival in the subgroup with non-paroxysmal AF.

There was no recurrence of atrial arrhythmia in 81.1% of the S-LGE group and 69.7% of the E-LGE group after ablation over 1 year of follow-up (p = 0.060). The estimated recurrence-free survival is shown in Fig. 3. The impact of LA-LGE extent on clinical outcome was prominent only in patients with non-PAF. Freedom from atrial arrhythmia occurred in 81% of the S-LGE group and 86% of the E-LGE group among patients with PAF (log-rank p = 0.529) and 81% of the S-LGE group and 55% of the E-LGE group in patients with non-PAF (log-rank p = 0.014). However high LA pressure (defined as LA pressure ≥ 21 mmHg) was not predictor of AF-free survival in this study regardless of AF type (Log rank p = 0.799 in overall, p = 0.562 in patient with PAF, p = 0.750 in patient with non-PAF) (Fig. S4).

## Discussion

This study showed that (1) the LA-LGE extent on CMR imaging was associated with clinical HF and elevated LA pressure and (2) the E-LGE group showed worse outcomes after catheter ablation, especially in patients with non-PAF.

CMR imaging in patients with AF has the advantage of providing an exact detailed image of the LA and surrounding structures. Furthermore, CMR imaging can assess the reservoir, conduit, and contractile function based on phasic changes in volumes. The LA-LGE in CMR images showed the degree of LA fibrosis. In addition, the LA-LGE extent in CMR imaging can non-invasively predict stroke risk^10^ and clinical outcomes of RFCA for AF^11^. Atrial fibrosis revealed by LGE-CMR imaging is useful in identifying optimal candidates for procedures to treat AF. These findings suggest that CMR imaging improved the selection process and outcome for patients considered for AF ablation. However, the clinical application of LGE-CMR imaging assessment is still limited because of poor reproducibility in different centers.

LA-LGE is a marker of atrial fibrosis^12,13^ and is correlated with the scar area in electrophysiologic studies of patients with AF. In our study, patients with extensive LGE (E-LGE group) had high rates of HF history, increased NT pro-BNP, and high LA pressure. These markers are associated with clinical heart function and atrial remodeling. Atrial fibrosis could either represent the cause or result of physiologic dysfunction. Habibi et al. showed that LA-LGE in feature-tracking CMR imaging was associated with LA function independent of age, sex, hypertension, type of AF, LA size, and wall thickness^14^. In the present study, the LA volume did not differ between the groups although it is a well-known marker of LA remodeling. Chrispin et al. reported that the pre-ablation LA-LGE extent was associated with increased LA volume. However, the strength of the relationship was weak in ablation candidates^15^. Early-stage remodeling with atrial fibrosis in LA did not show certain LA dilatation. We showed that atrial fibrosis was associated with the directly measured LA pressure. Increased LA pressure is a marker of diastolic dysfunction of the heart. Prior studies have hypothesized that atrial fibrosis is the result of atrial dilatation and diastolic dysfunction as evaluated by imaging studies, such as echocardiography or CMR.

### Association of LA-LGE and LA pressure

Increased LA pressure provokes the occurrence and maintenance of AF^16^. The LA pressure is the output of LV diastolic function, and LV diastolic dysfunction can increase atrial afterload. Peak LA pressure occurs during the early filling phase of the atrium. Atrial stretch and dilatation to compensate for the rising atrial wall stress subsequently result in AF^17^. The LA can compensate for these demands through the generation of more extracellular matrix, with the expression of proteins such as collagen. With increasing atrial wall stress, atrial fibrosis progressively creates a substrate for AF^18^. Hemodynamic stress, such as ventricular diastolic dysfunction, causes the development of fibrosis rather than hypertrophy in the atria since the thin-walled LA responds to pressure overload with dilatation rather than wall thickening^19^. Atrial fibrosis impairs inter-myocyte coupling via gap junctions, resulting in fragmented conduction that promotes arrhythmias^20^. Some prior reports have suggested a relationship between atrial stretch and AF substrates. LA pressure directly affects the maintenance of AF by mechanoelectrical feedback or stretch-induced pro-fibrotic reactions, creating AF substrates. LA pressure-independent atrial dilatation also increases the critical mass that begets AF.

### LA-LGE, a non-invasive marker of outcome after catheter ablation

The LA-LGE in CMR imaging offers a new predictor of clinical outcome after catheter ablation, reflecting LA anatomical remodeling with fibrosis. The current ablation strategy is mainly decided based on the clinical type of AF, i.e., paroxysmal or non-paroxysmal. However, LA remodeling with fibrosis did not closely correspond to the type of AF in this study. The non-PAF patients with low-grade LA-LGE showed good outcomes similar to those of PAF patients (more than 80% with AF/AT freedom rates over 1 year). A well-known LGE-CMR study in AF patients, DECAAF (Delayed-Enhancement MRI Determinant of Successful Radiofrequency Catheter Ablation of Atrial Fibrillation), also showed a weak correlation between AF type and LA-LGE^12,21,22^. The LA-LGE in CMR imaging reflects atrial anatomical and physiologic remodeling and may be a surrogate marker of the clinical AF type for ablation strategy decisions.

### Limitations

Our study was limited by its single-center, observational design. In addition, routine performance of quality 3D LGE-CMR imaging studies in patients requires a high level of expertise in CMR imaging and support during the studies by expert MR technologists and imagers. Processing of the CMR images is laborious and requires experienced observers to perform LA wall tracings and select the threshold levels. Improvements are expected to simplify and further standardize image processing. The accurate quantitative analysis of LA-LGE also remains a challenge. The walls of the LA are thin (1–2 mm) and susceptible to a partial volume effect from the surrounding compartments, like blood or the esophagus. To attenuate the inter-observer error of the quantification method, the LA-LGE volumes were measured twice by two independent investigators. LA pressure is not a fixed parameter because it is sensitive to the volume and heart rate of the patient. We measured the LA pressure at the beginning of the procedure after overnight fasting for 8 h. To prevent any impact from LA stunning, we measured the LA pressure 5 min after cardioversion. The catheter designed to carefully measure the pressure was not used. Finally, the image quality of LGE image in the quantification of LA wall composition is often confused by arrhythmia and it can impact the result of this study. We initially excluded the patients with unacceptable quality of CMR images. The impact of rhythm status during CMR to result was not considered because LGE amount and procedural outcome were not related to rhythm status during CMR in patients with PAF. In addition, the most non-paroxysmal AF patients underwent CMR during AF status.

## Conclusions

LA-LGE in CMR imaging was associated with LA remodeling and clinical HF. Increased LA pressure was related to the extent of LGE in the LA. LA-LGE was a good predictor of outcome after catheter ablation, but only in patients with non-PAF.

## Supplementary information

Supplementary Figures.
