# Supplementary material for: Association of left atrial pressure with late gadolinium enhancement extent in patient who underwent catheter ablation for atrial fibrillation
Source: Sci Rep. 2020 Oct 5;10:16486. doi: 10.1038/s41598-020-72929-0 (PMC7536288; doi:10.1038/s41598-020-72929-0)

**Association of Left Atrial Pressure with Late Gadolinium Enhancement Extent in Patient who Underwent Catheter Ablation for Atrial Fibrillation**

Seung-Young Roh, MD^1^, Dae In Lee, MD^2^, Sung Ho Hwang, MD, PhD^3^, Kwang-No Lee, MD^4^, Yong-soo Baek, MD^5^, Mohammad Iqbal, MD^6^, Dong-Hyeok Kim, MD^7^, Jinhee Ahn, MD^8^, Jaemin Shim, MD, PhD^4^, Jong-Il Choi, MD, PhD^4^, Young-Hoon Kim, MD, PhD^4^

**Figure S1.** The analysis process of the LA-LGE on basis of different tissue references. (**A)** Thin slice LGE-CMR image. **(B)** Segmentation of LA wall on LGE-CMR image. **(C)** Selection and quantification of LA wall LGE. **(D)** Reformatting of the 3D LA wall model showing entire LA wall LGE.

LA, left atrial; LGE, late gadolinium enhancement; CMR, cardiac magnetic resonance.


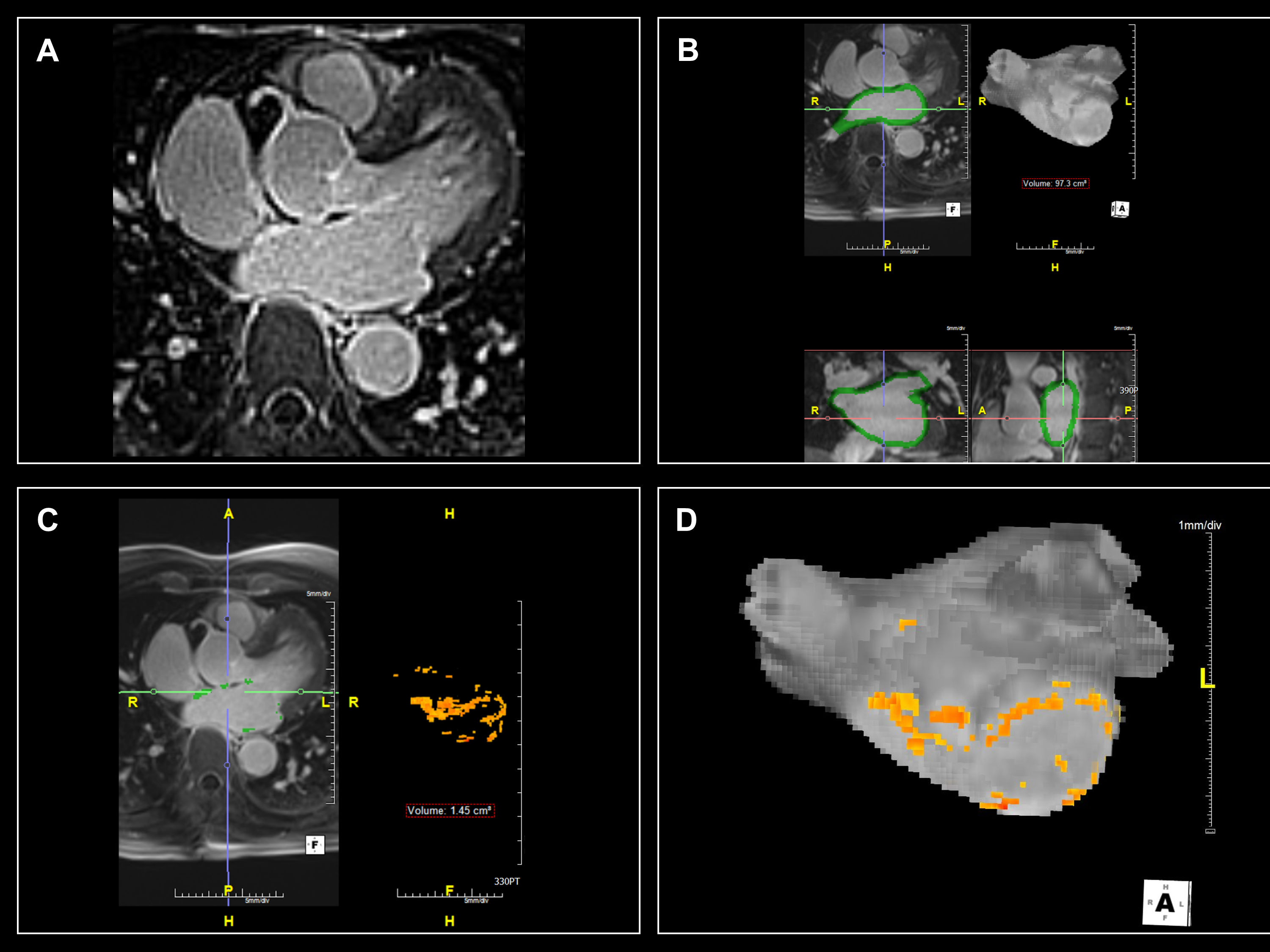


**Figure S2**. Measurement of left atrial (LA) pressure during sinus rhythm status

1. The pigtail catheter was inserted to the left atrium via a long sheath after septal puncture.
2. The typical curve of LA pressure measured during sinus rhythm status. It consisted of the wave (left atrial contraction), x wave (left atrial relaxation), v wave (left atrial filling), and y wave (left ventricle filling). Pressure at the v wave (asterisk) was defined as the LA pressure.

RA, Right atrium; LA, Left atrium; ECG, Electrocardiography.


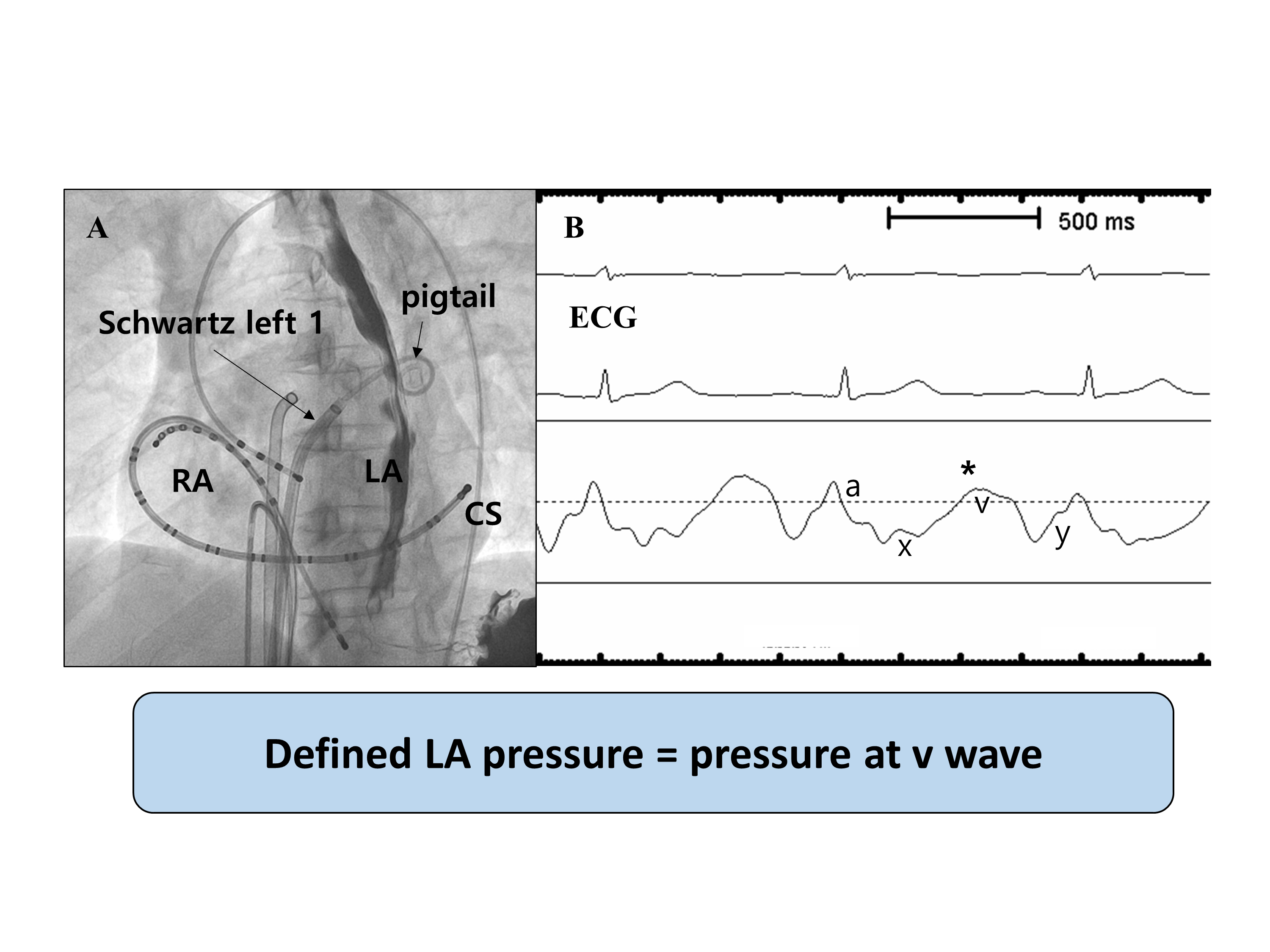


**Figure S3.** Measured value of serum biomarkers in both groups. E-LGE group have higher NT pro B-type natriuretic peptide level compared to S-LGE group.

BNP, NT pro B-type natriuretic peptide; vWF, von Willebrand factor; hs-CRP, high-sensitivity C-reactive protein.


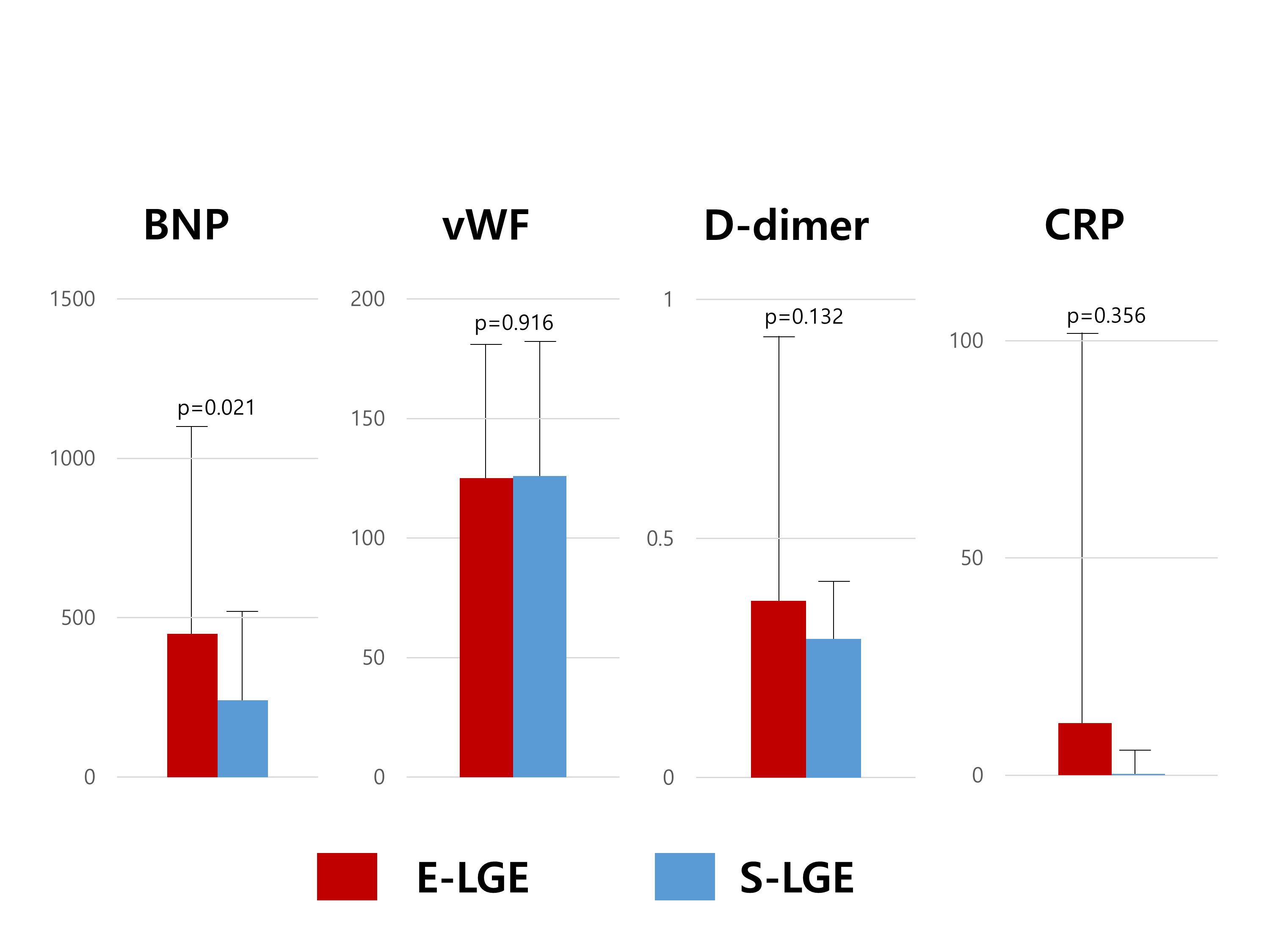


Figure S4. Kaplan–Meier survival curve showing atrial fibrillation (AF)/atrial tachycardia (AT)-free survival with the proportion of patients in sinus rhythm against the follow-up time in days along left atrial pressure. (A) AF/AT-free survival in the total population, (B) AF/AT-free survival in the subgroup with paroxysmal AF, (C) AF/AT-free survival in the subgroup with non-paroxysmal AF.

AF, atrial fibrillation; LAP, left atrial pressure; high LAP was defined as LAP ≥21mmHg, median index of LAP of overall study population. Low LAP was defined as LAP <21mmHg.


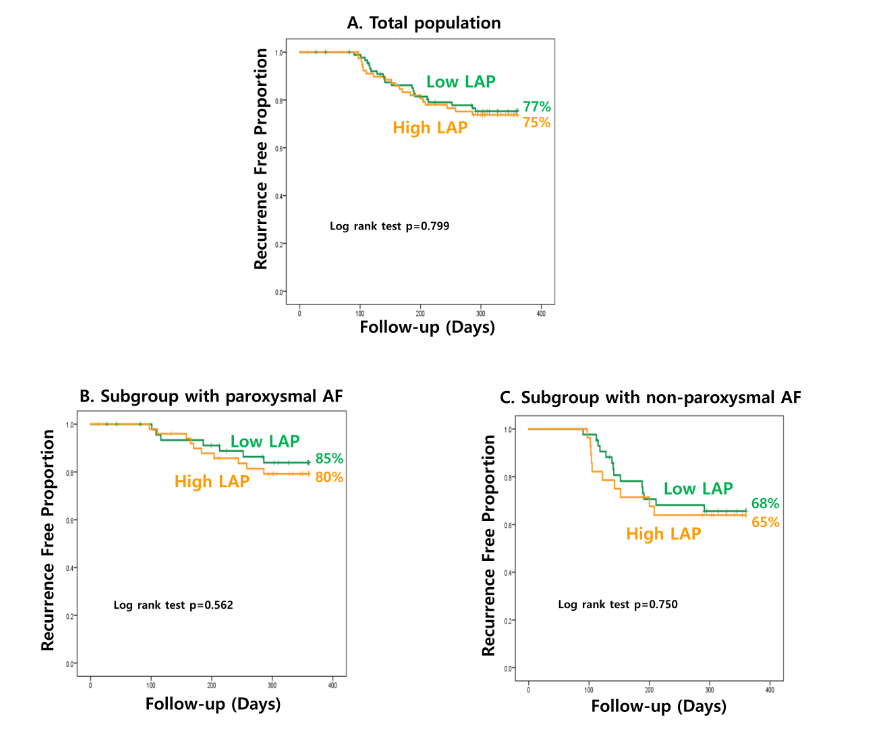

Supplement: Supplementary file 1 — Supplementary Figures. [file 41598_2020_72929_MOESM1_ESM.docx]
